# Promoting Long-Term Health among People with Spinal Cord Injury: What’s New?

**DOI:** 10.3390/ijerph14121520

**Published:** 2017-12-06

**Authors:** Mary Ann McColl, Shikha Gupta, Karen Smith, Alexander McColl

**Affiliations:** 1 School of Rehabilitation Therapy, Queen’s University, Kingston, ON K7L 3N6, Canada; 15sg48@queensu.ca; 2 Providence Continuing Care Centre, Kingston, ON K7L 4X3, Canada; smithk2@providencecare.ca; 3 Department of Family Medicine, University of New South Wales, Port Macquarie, NSW 2444, Australia; mccoll4@bigpond.net.au

**Keywords:** spinal cord injury, primary care, health promotion, evidence-based care, scoping review, primary physicians

## Abstract

A key ingredient to successful health promotion is a primary care provider who can offer an informed first response to lifestyle issues, emerging problems and chronic challenges. This article aims to assist family physicians to play their role in promoting the health of people with SCI, by summarizing the latest evidence in the management of spinal cord injury in primary care. This study used a scoping review methodology to survey peer-reviewed journal articles and clinical guidelines published between January 2012 to June 2016. This search strategy identified 153 articles across 20 topics. A prevention framework is used to identify five primary, nine secondary, four tertiary, and two quaternary prevention issues about which family physicians require current information. Major changes in the management of SCI in primary care were noted for 8 of the 20 topics, specifically in the areas of pharmacological management of neuropathic pain and urinary tract infection; screening for bowel and bladder cancer; improvements in wound care; and clarification of dietary fibre recommendations. All of these changes are represented in the 3rd edition of *Actionable Nuggets*—an innovative tool to assist family physicians to be aware of the best practices in primary care for spinal cord injury.

## 1. Introduction

A key ingredient to successful health promotion is a good relationship with a primary care provider who can offer an informed first response to lifestyle issues, emerging problems, and chronic challenges. For more than 85,000 Canadians living in the community with a spinal cord injury (SCI) [1], family physicians are essential for promoting health and managing disability [2]. As people with SCI live longer with potentially greater burdens of chronic disease, family physicians assist with screening for life-threatening illness, preventing secondary conditions, and promoting a healthy lifestyle [3]. 

People with SCI, however, often find that their family doctor does not fully appreciate the complexity of their situation, or the limitations their disability imposes on the options for health promotion and care [4]. Family physicians themselves report that managing patients with disabilities like spinal cord injury is challenging [5,6,7]. Although the prevalence of spinal cord injury is low (often only one or two in a caseload), the complexity of care is high.

The main difficulty for people with SCI is to be confident that their family physician is equipped with the latest evidence and options [8,9]. To address this challenge, an innovative tool called *Actionable Nuggets* was developed to provide best evidence to family physicians about health promotions and primary care for people with spinal cord injury. *Actionable Nuggets* are brief, focused communications in the form of postcards about the needs of special populations in primary care. Each *Nugget* consists of approximately 300 words, including:

- A clinical problem;
- The *Actionable Nugget* itself—i.e., a specific recommendation for practice;
- A description of best practice and the evidence supporting it;
- A key reference for the recommendation;
- A link to the website for further resources—typically 40–100 additional references per topic (www.actionnuggets.ca).

The development of *Actionable Nuggets* began with a scoping review in 2009 to identify the most pressing long term health issues seen in primary care in people with SCI. The search identified 20 topics, and 20 *Actionable Nuggets* were developed using topic-specific literature searching and exhaustive review by an Expert Panel [10]. The original *Actionable Nuggets* were produced in both hard copy and electronic post cards. The 20 postcards were disseminated weekly to a sample of family physicians from Ontario, Newfoundland and Australia. Data were collected regarding changes in knowledge, attitudes and practices, as well as feedback on the utility and acceptability of *Nuggets*. The results of the evaluation showed that this evidence based resource was an excellent fit with information needs and consumption processes in primary care [10]. Family physicians appreciated that *Nuggets* helped address complex or unknown topics, that they synthesized a large body of literature, and that they could be consumed in about 1 min—literally while walking between the mailbox and his desk, according to one subscriber!

In 2012, the 2nd edition of SCI *Nuggets* was produced and distributed to approximately 50,000 physicians in Canada through the electronic continuing education portal of the Canadian Medical Association, in both English and French. *Nuggets* were accredited by the College of Family Physicians of Canada for 10 M1 Mainpro credits. There were almost 8000 views through the CMA site, of whom 89% were family physicians and 11% were specialists [11]. Around 57% reported that they learned new information, and 43% said that the *Nuggets* either confirmed what they were already doing or reminded them of something that they knew. In total, 86% stated that they were very satisfied with the content and presentation of information, and 43% said that the information was directly relevant to at least one of their patients.

Currently the 3rd edition of *Actionable Nuggets* has been launched for distribution through the *Actionable Nuggets* website (www.actionnuggets.ca). Each revision of *Nuggets* has resulted in numerous changes to the content, based on the latest evidence. Some of those changes are minimal, and some are significant, with major implications for health maintenance and long-term health. 

The purpose of this article is to assist family physicians to play their role in promoting the health of people with SCI, by summarizing the changes in the management of spinal cord injury in primary care that have arisen in the literature between the 2nd & 3rd editions (2013–2016). 

## 2. Materials and Methods

This study used 20 topic-specific scoping reviews to identify new information for each topic pertaining to primary care of people with spinal cord injuries [12,13]. Peer-reviewed journal articles, reviews and clinical guidelines were searched according to the following inclusion criteria:

- published between January 2012 to June 2016;
- searched using Pubmed, OVID, CINAHL;
- available in English.

The keywords used for each search, in addition to those listed here are provided in Table 1: spinal cord injury, paraplegia, quadriplegia, tetraplegia AND primary care, family medicine, community care, chronic disease management, family physician, general practice, long term care.

This search strategy identified 21,783 articles. After excluding duplicates, two reviewers screened the abstracts of each article, and articles were excluded if they did not meet the following criteria:

- primary focus on conditions occurring one or more years post spinal cord injury;
- sample size greater than five;
- community (vs. institutional/inpatient) care;
- focus on secondary health conditions associated with SCI;
- relevant to the primary health care setting.

The resulting 238 articles were read in their entirety. By consensus, the authors excluded an additional 85 articles that did not provide sufficient evidence to warrant a change in practice. Thus, the final number of included articles was 153 (see Table 1). These were sorted according to the 20 Nuggets topics, and findings extracted for discussion by the Expert Panel. 

The Expert Panel consisted of five experts in family medicine, rehabilitation, and knowledge translation. Through three rounds of revision using a consensus-seeking process, the pertinent articles for each Nugget were reviewed, and the evidence was evaluated for its implications for practice. Both qualitative and quantitative evidence were considered, using standard critical appraisal techniques. Where indicated, the content of each Nugget was revised to reflect the best evidence available as of September 2016. Prior to being considered final, each Nugget had to be approved consensually by all members of the Panel. In some instances, many rounds of reviewing were required before the Panel was prepared to sign off. 

## 3. Results

The results are presented based on the 20 key topics of Nuggets. We highlight the changes made in each of the Nuggets, reflecting the latest evidence or changes in clinical practice guidelines that have emerged in the last three years. 

### 3.1. Minor Changes

Minor changes were recommended for 12 Nuggets, as shown in Table 2 and discussed below:

*Nugget #1—Epidemiology of Spinal Cord Injury*—The incidence of spinal cord injury continues to be estimated at 3–5/100,000, and the leading causes of premature death are respiratory or cardiovascular [1].

*Nugget #2—Screening for Cardiovascular Risk*—The National Cholesterol Education Program’s Adult Treatment Protocol III is still recommended as a most accurate classification of lipid levels for the SCI population, as the Framingham Risk Score has been shown to underestimate CVD risk in the SCI population [14].

*Nugget #3—Management of Cardiovascular Risk*—Physical activity guidelines recommending ≥20 min of moderate to vigorous aerobic exercise twice a week, along with strengthening, continue to represent best practice. One line was added referring to recent studies suggesting that cognitive deficits may result from hypotension (lesions above T1) and arterial stiffness (lesions below T7) [15].

*Nugget #4—Autonomic Dysreflexia*—The standard for practice remains the same for AD, with one exception. The increased use of PDE5i (such as Viagra, to enhance sexual function) warrants a warning against the use of nitrates in patients using these drugs [16].

*Nugget #5—Assessment of Pain in SCI Patients*—The Douleur Neuropathique en 4 Questions (DN4) questionnaire continues to be the screening tool of choice for distinguishing between neuropathic and musculoskeletal pain. Psychometric values have been updated—83% sensitivity and 90% specificity [17].

*Nugget #7—Management of Musculoskeletal Pain*—A team approach continues to be the optimal way to manage musculoskeletal pain in the upper extremity, back and neck, with surgery an option when conservative measures fail [18]. 

*Nugget #8—Annual assessment of Neurogenic Bowel*—Annual bowel assessment remains essential for patients with neurogenic bowel, to meet the goals of continence, reasonable evacuation time and regularity [19]. 

*Nugget #9—Evaluation of Bowel Management Program*—A step-wise approach to bowel management in primary care remains the best course of treatment, from conservative techniques to pharmacological measures, to surgical intervention [20]. The revised Nugget acknowledges new methods of bowel management, such as trans-anal irrigation and nerve root stimulation.

*Nugget #12—Routine Monitoring of Neurogenic Bladder*—The revised Nugget clarifies the importance of annual bladder monitoring in primary care including bloodwork and ultrasound, as well as periodic cystoscopy and urodynamic assessment with a urologist [21]. There is a lack of strong evidence on the optimum follow up schedule for urodynamics [22]. 

*Nugget #16—Prevention of Skin Breakdown*—Although there are no conclusive guidelines to prevent pressure injuries, an individualized interdisciplinary approach continues to be recommended, with the Braden scale as the gold standard for assessing risk [23]. 

*Nugget #18—Depression and SCI*—Screening for depression (using the Patient Health Questionnaire [PHQ-9]) continues to be an important role in primary care with people with spinal cord injuries, given incidence rates 2–4 times greater than those in the general population [24]. 

*Nugget #19—Sexuality in Spinal Cord Injury*—Sexual activity, sexual function and reproductive issues often arise post-rehabilitation, and the family physician is the best person to ensure that conversations take place and the appropriate resources are sought [25]. 

### 3.2. More Substantive Changes

Substantive changes were indicated for eight of the 20 Nuggets in the 3rd Edition. These Changes Are summarized below and presented in Table 3:

*Nugget #6—Pharmacological Management of Neuropathic Pain*—The stepwise protocol for therapeutic agents to treat neuropathic pain has been altered. Instead of gabapentinoids alone as first-line treatment, this category now also includes tricyclic antidepressants and serotonin-noradrenalin re-uptake inhibitors (SNRIs), which work equally well for some patients [26,27,28]. Consequently, controlled-release opioids become the second-line treatments, cannabinoids the third, and a number of other drugs, such as methadone, other anti-convulsants, and botulinum toxin are fourth [29]. Evidence is emerging for physical modalities, such as physical and electrical stimulation [30,31,32,33].

*Nugget #10—Diet and Fluid Management in Neurogenic Bowel*—New guidelines are offered for diet and fluid management with neurogenic bowel. 15–18 g of fibre per day is recommended, along with 35 mL of fluid (preferably plain water) per kilogram of body weight. This results in a daily intake about 500 mL higher than the general population [34]. Fluid intake must be modulated as it will have consequences for bladder emptying. 

*Nugget #11—Screening for Colorectal Cancer*—Recommendations for colorectal cancer screening are the same for patients with or without neurogenic bowel [35]. These recommendations have changed quite significantly. They recommend ceasing screening at 74 years of age for those without significant family history, and screening half as often. Specifically, they suggest fecal occult blood test (FOBT) every 2 years, or flexible sigmoidoscopy every ten years. For individuals over 75 years of age screening is not recommended. Further, colonoscopy is not recommended as a screening tool, but rather as a definitive diagnostic following a positive screen [36]. The new Nugget acknowledges the very significant ordeal of colonoscopy preparation for individuals with neurogenic bowel, and the potential for compromised results due to incomplete bowel emptying [37]. 

*Nugget # 13—Recognizing Urinary Tract Infections (UTI) in SCI Patients*—Diagnosis of UTI in SCI continues to rely on three criteria: (1) significant bacteriuria; (2) pyuria; AND (3) signs and symptoms; however, the most recent guidelines increase the concentration of cells for bacteriuria: ≥10^5^ cfu/mL (vs. 10^2^ in 2013) for those using intermittent catheterization; ≥10^7^ cfu/mL (vs. 10^4^) for those using condom drainage; and ≥10^8^ cfu/mL (vs. 10^4^) for those with spontaneous bladder emptying [38]. Recent evidence also suggests that urine dipstick testing may prove as accurate as microscopy [39]. 

*Nugget #14—Pharmacological Management of UTI in SCI*—Nugget # 14 is one of two where the actual recommendation changed. Whereas the 2nd ed. (2013) explicitly recommended fluoroquinolones as the first-line treatment for UTI with SCI, recent literature indicates that there is no superior agent or class of antibiotics. In fact, there is no evidence-based standard of care for urinary tract infections in SCI [38,40]. Instead, the recommendation emphasizes the need to match antimicrobial agents to the infecting organism and the patient’s risk profile [41]. A recent survey showed poor compliance in matching culture sensitivity with antibiotic prescribed and a tendency to over-treat UTIs with SCI, resulting in antibiotic resistance [42,43]. The new Nugget also distinguishes simple (frequency of fewer than three per year) from complicated (>3 infections per year) infections, and recommends a short course treatment (3–7 days) for the former, and long-course (7–14 days) for the latter [38].

*Nugget* # 15—Screening for Bladder Cancer in SCI Patients—As of the 3rd edition of Nuggets, routine screening for bladder cancer is no longer recommended for all SCI patients, but only for those considered high-risk—that is, those with indwelling or suprapubic catheters, complete lesions, >10 years since injury, bladder stones, or recurrent urinary tract infections [44]. Cystoscopy is still the best screening tool; however, given the relatively low prevalence of bladder cancer with SCI (<2%), there is controversy about the benefits of screening [44,45]. 

*Nugget # 17—Treatment of Skin Breakdown*—Changes to Nugget # 17 reflect the wide array of emerging treatment options for pressure injuries. A number of dressing options are available, including hydrocolloid, transparent film, hydrogel, alginate, foam, silver, silicone, collagen matrix, honey and iodine [46]. Biophysical modalities, such as electro-magnetic agents, pulsed radio frequency, phototherapy, ultrasound, electrical stimulation and negative pressure therapy are also accumulating evidence [46]. Collaborative multidisciplinary management continues to be recommended, including physical and occupational therapy, nursing, nutrition and rehabilitation specialists. Prompt referral to a surgeon is recommended for Stages III and IV wounds, in order to prevent osteomyelitis.

*Nugget # 20—Wheelchair Accessibility of your Practice*—The new Nugget # 20 reflects the changing legal environment with regard to accessibility. It is now the law in a number of jurisdictions that private practices are required to be accessible [3]. The simple addition of an adjustable examining table and ceiling-track lift in one examining room benefits not only SCI patients, but many other patients in the practice as well [47].

## 4. Discussion

This article aims to ensure the efficacy of a key ingredient in health promotion for people with spinal cord injury, by ensuring a high standard of primary care. Specifically, the paper summarizes the latest changes in evidence-based primary care for people with spinal cord injuries. It focuses on the 20 most important health issues presenting in primary care among people with SCI, according to a scoping review of the international peer-reviewed literature [10,11,48]. These 20 topics range from those that would be relatively common in the primary care setting to those that would be clearly outside the usual routine. In applying the content of these 20 topics to health promotion, it is useful to think in terms of the four levels of prevention:

- primary prevention targets the whole population, with the aim of preventing exposure to risk factors;
- secondary prevention targets those already identified at risk, with the aim of preventing illness or injury;
- tertiary prevention targets those who are already ill, with the aim of preventing disability;
- quaternary prevention targets those already disabled, with the aim of preventing social disadvantage [49].

Some might argue that all health promotion among disabled people is quaternary prevention, due to the presence of a pre-existing disability; however, if we consider people with spinal cord injuries as the population, then the prevention levels offer a useful framework for understanding the health promotion needs of people with spinal cord injury, as shown in Table 4. 

Among the 20 topics covered by *Actionable Nuggets,* five represent primary prevention, nine secondary prevention, four tertiary prevention, and two quaternary prevention.

- Primary prevention includes topics that attempt to maintain the health of people with SCI, and to prevent excess risk of non-SCI-related comorbidities; for example, basic epidemiology of the main health challenges in the SCI population, access to health services, sexual and reproductive health, and screening for cardiovascular risk and bowel cancer.
- Secondary prevention in spinal cord injury includes the prevention of those illnesses and complications for which the person might be particularly at risk by virtue of having a spinal cord injury. Some examples include the management of:
  ◯ cardiovascular risk, which is known to be elevated in SCI;
  ◯ autonomic dysreflexia, a condition peculiar to those with lesions above T6;
  ◯ neurogenic bowel and bladder, and screening for bladder cancer in high risk patients;
  ◯ skin lesions that might arise in sensation-impaired regions;
  ◯ depression, also significantly elevated particularly early after onset.
- Tertiary prevention refers to those situations where a comorbid condition or secondary complication has also arisen, and the role in primary care is to prevent it from causing further disability or compromise to functional independence. These topics often require the input of a specialist, and they include the management of:
  ◯ Pain, whether neuropathic or musculoskeletal;
  ◯ Urinary tract infections.
- Quaternary prevention refers to situations where the individual already has acquired a secondary disability, due to issues like pain or skin breakdown. These two topics typically require the participation of multidisciplinary team to ensure that quality of life is not further unduly compromised.

In 12 of the topics covered, only minor changes were required to update the *Nuggets* and provide the latest evidence-based information to primary care practitioners. In 8 of the topics, significant change to the *Nugget* was required to reflect the latest and best evidence in the literature, emerging between 2013 (2nd ed.) and 2016 (3rd ed.). The main changes to practice involve:

- Pharmacological management of neuropathic pain and urinary tract infection;
- Screening for bowel and bladder cancer;
- Improvements in wound care; and,
- Clarification of dietary fibre and fluid recommendations.

In addition, changes to the statutory environment in some jurisdictions prompted changes to the recommendations about office or clinic accessibility for people with wheelchairs and other disabilities.

## 5. Conclusions

The 3rd edition of *Actionable Nuggets* summarizes the latest evidence on primary care for people with spinal cord injuries. The 20 topics covered are of significant for this small but complex population. Notable changes in the standard of care between 2013–2016 are summarized in this paper. These patients depend heavily of the family physician for all levels of preventive care—to recognize emerging complications and provide timely and effective care. *Actionable Nuggets* (3rd ed.) reflect ‘best practice’ at this time, and are available free of charge at www.actionnuggets.ca.

## Figures and Tables

**Table 1:** List of Nuggets and keywords used for scoping review.

Nugget #	Key Words Used in Addition to Those Listed above	Total # Articles Screened	Total # Reviewed	Final # Included
*1. Epidemiology of spinal cord injury*	epidemiology, incidence, prevalence, burden, complications, causes of death, co-morbidities	49	13	5
*2. Screening for cardio-vascular risk in SCI*	cardiovascular, complications, screening, assessment	618	10	4
*3. Management of cardio-vascular risk in SCI*	cardiovascular, complications	618	10	10
*4. Autonomic dysreflexia*	autonomic dysreflexia	411	21	14
*5. Assessment of Pain in SCI Patients*	pain, assessment	6705	15	11
*6. Pharmacological Mgt. of Neuropathic Pain*	pain, pharmacological management, neuropathic	338	14	13
*7. Management of Musculoskeletal Pain*	pain, musculoskeletal, soft tissue	937	4	4
*8. Annual Assessment of Neurogenic Bowel*	neurogenic bowel	841	4	3
*9. Periodic Re-evaluation of Bowel Management*	neurogenic bowel	841	15	11
*10. Diet & Fluid Management in Neurogenic Bowel*	diet, fluid, management, constipation	152	4	4
*11. Screening of colorectal cancer*	colorectal cancer, neoplasms, bowel	227	21	5
*12. Routine Monitoring of neurogenic bladder*	neurogenic bladder	384	22	16
*13. Recognizing UTI in SCI patients*	urinary tract infection	2860	20	10
*14. Pharmacological management of UTI in SCI*	urinary tract infection, drug, pharmacology, antibiotic	1288	13	11
*15. Screening of bladder cancer in SCI patients*	urinary, bladder, neoplasms, cancer	388	5	4
*16. Prevention of skin breakdown*	skin breakdown, pressure ulcer	1259	11	7
*17. Treatment of skin breakdown*	skin breakdown, pressure ulcer	805	12	5
*18. Depression and SCI*	depression	1747	6	5
*19. Sexuality in SCI*	sexual dysfunction	1212	12	8
*20. Wheelchair accessibility of your practice*	accessibility, wheelchair, universal design	103	6	3
TOTAL		21,783	238	153

“#” represents number.

**Table 2:** 12 Nuggets with minor modifications.

#	Nugget Topic	Nugget Content (3rd ed.)
1.	*Epidemiology of SCI*	Be aware of the most important health risks for patients with spinal cord injuries.
2.	*Screening for cardiovascular risk*	Screen for cardiovascular risk factors at least annually.
3.	*Management of cardiovascular risk*	Manage cardiovascular risk among patients with SCI as you would a high-risk ambulatory patient.
4.	*Autonomic dysreflexia*	Alert patients with SCI at T6 & above to the risks of AD, and the need for urgent treatment at the onset of an episode.
5.	*Assessment of pain in SCI patients*	Distinguish between neuropathic and musculoskeletal pain in your patient with SCI, and monitor pain regularly.
7.	*Management of musculoskeletal pain*	Chronic musculoskeletal pain requires an interdisciplinary approach, including rehabilitation, and in some cases, surgery.
8.	*Annual assessment of Neurogenic bowel*	Conduct an annual assessment of bowel function in patients with SCI.
9.	*Evaluation of bowel management program*	A step-wise approach to bowel management is recommended, with the involvement of SCI specialist as needed.
12.	*Routine monitoring of neurogenic bladder*	Bladder function should be reviewed annually by the family physician, and periodically by a urologist.
16.	*Prevention of skin breakdown*	Assess for risk of pressure injuries using the Braden Scale, and refer to rehabilitation specialist if high risk.
18.	*Depression and SCI*	Screen for depression annually in patients with SCI, using the PHQ-9 or PHQ-2, and treat at standard guideline levels.
19.	*Sexuality in SCI*	Sexual activity, sexual function and reproductive issues should be addressed as part of an annual examination.

“#” represents number.

**Table 3:** 8 Nuggets with major modifications.

#	Nugget Topic	Nugget Content (3rd ed.)
6.	*Pharmacological management of neuropathic pain*	Use a step-wise evidence-based protocol for the management of neuropathic pain in SCI, and review pain management annually.
10.	*Diet & fluid management in neurogenic bowel*	Refer spinal cord injured patients with persistent constipation to a specialist with experience with SCI or neurogenic bowel.
11.	*Screening of colorectal cancer*	Initiate colorectal cancer screening for patients with SCI using the same principles as those for the general population.
13.	*Recognizing Urinary Tract Infections in SCI Patients*	Diagnosis of UTI in SCI patients requires 3 criteria: (1) significant bacteriuria; (2) pyuria; and (3) signs & symptoms.
14.	*Pharmacological management of UTI in SCI*	There is no superior agent or class of antibiotics for UTIs in spinal cord injury. Recurrent UTIs should be treated as complicated infections, and treatment must be customized to the patient and the infecting organism.
15.	*Screening for Bladder Cancer*	Routine screening for bladder cancer is recommended only for high-risk patients; that is those with indwelling or suprapubic catheters, complete lesions, >10 years since injury, bladder stones, or recurrent urinary tract infections.
17.	*Treatment of Skin Breakdown*	Treat Stage I or II wounds with standard wound care. Treat Stage III or IV wounds with specialist/surgical intervention.
20.	*Wheel chair accessibility of your practice*	Conduct an accessibility audit of your office or clinic space and procedures using the Primary Care Accessibility Checklist.

“#” represents number.

**Table 4:** Goals with respect to levels of prevention in SCI population.

Level of Prevention	Focus in the General Population	Focus in the SCI Population	Goal in the General Population	Goal in the SCI Population	Nuggets/Topics
Primary	The entire population	Otherwise healthy people with uncomplicated SCI	Prevent exposure to risk factors	Prevent exposure to additional risk	1, 2, 11, 19, 20
Secondary	Those at risk	Those with higher risk of certain complications	Prevent illness	Prevent secondary illness & complications	3, 4, 8, 9, 10, 12, 15, 16, 18
Tertiary	Those who are ill or injured	Those who already have a particular complication or comorbidity	Prevent disability	Prevent additional disability	5, 6, 13, 14
Quaternary	Those who are disabled	Those with additional secondary disability	Prevent social disadvantage	Prevent further compromise to QOL	7, 17
